# Exploring the challenges experienced by patients and families using palliative and end-of-life care services: A qualitative focus group study

**DOI:** 10.1017/S1478951525000057

**Published:** 2025-03-03

**Authors:** Gina Kallis, Gary Hodge, Hannah Wheat, Tomasina M. Oh, Susie Pearce

**Affiliations:** 1 School of Nursing and Midwifery (Faculty of Health), University of Plymouth, Plymouth, United Kingdom; 2 Peninsula Medical School (Faculty of Health), University of Plymouth, Plymouth, United Kingdom

**Keywords:** End-of-life, palliative care, patient experience, family carers, emotions

## Abstract

**Objectives:**

In the UK, access to dignified and compassionate palliative care is increasingly being reported as inadequate. This is a particular issue in South-West England, where there is an ageing population, historical lack of research, and particular challenges due to its rural and coastal location. The aim of this study was to provide a holistic view of patient and family experiences of care toward the end-of-life in this location and to collaboratively identify the challenges they face.

**Methods:**

Six qualitative focus groups were held with people who were toward the end of their life, family/carers, and people who were bereaved. Participants were recruited via hospice networks. Most focus groups were face-to-face, and all were facilitated by two researchers.

**Results:**

A range of challenges were identified at different stages of the patient and family carer journey near the end-of-life. These included issues related to the delivery of care, such as communication challenges, a lack of out of hours care, and also a lack of personalized care. Patients and families also experienced everyday challenges due to the impact of living with a life-shortening condition and altered family dynamics as family members became carers. There were also some traumatic experiences of death and a sense of abandonment when care could not be accessed.

**Significance of results:**

This study provides a core first step in developing partnership working with members of the public, which can be built upon to develop future research agendas. This work has identified areas for improvement, so that ultimately, end-of-life experience for the individual, their carers, and families can be improved.

## Introduction

Palliative care refers to the holistic care of people living with life-threatening illness and includes physical, psychological, social, and spiritual support (Pask et al. 2022). Its aim is to relieve suffering and improve quality of life for both patients and informal carers. In the UK, palliative and end-of-life (EoL) care in the last 12 months of life is provided by a range of professionals across hospital, community, and hospice settings. However, access to dignified and compassionate end-of-life care in the UK is frequently considered inadequate (NHS England 2021; Sleeman et al. 2021). The National Institute for Health Research has identified this research area as a priority (NIHR 2022), and such research is sparse in the South-West of England. This is a concern, given the ageing population in this region; in 2018, the South-West Peninsula (Devon, Cornwall, and Somerset) had the highest percentage of population aged 65 and above compared to other areas in England (Hansford et al. 2023). Additionally, the area is largely rural with an extensive coastline. Research has found that those living in rural and coastal areas face challenges accessing healthcare, and these are exacerbated at the end-of-life (Hospice UK 2021).

Despite the inequalities experienced by people at the end of their lives, they are underrepresented in health and medical research (Johnson et al. 2021). In the UK, there is a small body of work which explores the experiences and perspectives of patients with a life-shortening condition (Almack et al. 2012; Conner et al. 2008; Costello 2001; Janssen and MacLeod 2010; Law 2009; Mason et al. 2022; Mayland et al. 2021; Payne et al. 2010; Reeve et al. 2012; Worth et al. 2006). Research has explored the complexities surrounding communication between patients and healthcare professionals in planning for EoL care (Almack et al. 2012; Reeve et al. 2012), and the need for personalized care (Reeve et al. 2012) which attends to patients’ emotional needs, as well as physical (Law 2009). Studies in Scotland have investigated palliative patients’ experiences of accessing and using out-of-hours healthcare services. Studies have shown a need for clearer information on how to access these services (Mason et al. 2022) and for these services to consider the complex needs associated with palliative and EoL care (Worth et al. 2006).

In this paper, we build on these studies and attempt to provide a holistic view of patient and family experiences of care toward the end-of-life in the South-West of England. The findings presented are part of a wider study which also included focus groups with healthcare professionals. However, this paper only reports the experiences of patients, family carers, and bereaved family members.

## Methods

### Data collection and participants

This was a qualitative study which explored people’s experiences of care near the end-of-life. Eighteen participants took part in six focus groups in June/July 2023. Potential participants were invited to participate by six hospices in the South-West of England. Those who expressed an interest in taking part in the study were contacted by a member of the research team or a key contact from the hospice. Focus groups took place on site at the hospices, except for one which was held at a local community center at the request of the hospice. All sessions were facilitated by two experienced qualitative researchers, including one with a nursing background in EoL care. Written consent was obtained before the focus groups began. A focus group guide (Appendix 1) was used, which asked participants about their experiences of palliative and EoL care in relation to either themselves or someone they knew, things they found both helpful and challenging, and what areas they viewed as a priority for future research. The approach was reflexive and conversational and facilitated qualitative, in-depth discussion (Barbour 2010).

Three focus groups were held with patients who were toward the end of their life and their family members/carers, and the other three were held with bereaved family members (Table 1). Focus groups had between one and seven participants; the focus group with only one participant was adapted to an in-depth interview. All sessions lasted between 75 and 110 minutes. They were audio recorded and field notes were captured immediately afterwards.

**Table 1.** Participants

Focus Group	Focus Group Type	Number in attendance	About participants
1	Patient and family carer	2 participants	One patient who was diagnosed with Motor Neurone Disease (MND) in December 2022 and was receiving palliative care from the hospice.
			The wife of the above patient also attended.
2	Bereaved family members	5 participants	A husband whose wife had died in August 2022.
			A husband and wife whose daughter had died in 2022.
			A husband whose wife had died in November 2022.
			A wife whose husband had died in February 2022.
3	Bereaved family member	1 participant	A wife whose partner had died several years prior.
4	Bereaved family members	2 participants	A family member whose partner had died in August 2016.
			A husband whose wife had died in November 2022.
5	Patient	1 participant	A patient with secondary breast cancer. Had initial diagnosis in 2008.
6	Patient and family carers	7 participants	One patient who began palliative care in July 2023.
			One patient diagnosed with cancer in 2018.
			One patient with cancer and his partner. He was diagnosed in 2022. Was receiving treatment for a tumor.
			A wife whose husband was diagnosed with kidney cancer in November 2022.
			One patient who was diagnosed with pancreatic cancer in March 2021.
			One patient who was diagnosed with cancer in May 2022.

### Ethics

Ethical approval was obtained from the University of Plymouth’s Faculty Research Ethics and Integrity Committee. All participants received a Participant Information Sheet and provided written informed consent before participating.

### Analysis

All recordings were professionally transcribed verbatim and anonymized. Transcripts were then uploaded to NVivo 12 software for analysis. An iterative approach to analysis was undertaken that followed the principles of reflexive thematic analysis (Braun and Clarke 2019; Braun et al. 2014). This combined both a deductive approach informed by the topic guide and an inductive approach, whereby codes were derived from the data. To enhance rigor, a team of five researchers independently analyzed one/two transcripts each, generating a selection of codes. Three of the researchers then reviewed all the codes. Following discussion, these codes were integrated into themes which were: communicating the diagnosis of a life-shortening condition; the impact of living with a life-shortening condition; challenges to patient and family carer relationships; a sense of abandonment when care is not provided/cannot be accessed; a lack of personalized care; feeling prepared for death; traumatic experiences of death; inconsistencies in the provision of bereavement support adding to the sense of abandonment. The themes are presented in the findings section across the different stages of the patient and family carer journey near the end-of-life.

## Findings: the journey through palliative and EoL care

### Entry into palliative care

#### Communicating the diagnosis of a life-shortening condition

Some individuals spoke about the way a life-shortening diagnosis was communicated to themselves or family members. In some instances, individuals were told their diagnosis when they were alone and without any family members present, or over the telephone:

*We saw the surgeon … he got us together – my daughter and my son and me – and said, “I’m going to operate but … I can save her life, but I can’t cure what it is.” I still had no inkling of what it was. So, the next day, the surgeon had already spoken to my wife who was on her own and told her that she had two months to live.* (Bereaved husband, Focus Group 2)

There were times where both patients and family members felt that more information would have been helpful regarding how the illness could potentially evolve and its symptoms, as the following focus group excerpt demonstrates:

Patient A: *As far as palliative care goes, I think one of the priorities should be sufficient information about what is likely to happen.*

Patient B: ‘*Cause you don’t actually get told very much at all, do you? “It’s come back. There’s nothing we can do” … And what, what things are insurmountable. And, and therefore how you cope with the corresponding, you know, perhaps pain or, or whatever it is that you’re going to experience.* (Focus Group 6)

It was often difficult to absorb this news and subsequent information, therefore the timing of its delivery was critical. Some participants felt that more training and education would be helpful for healthcare professionals to improve how and when they conducted challenging conversations.

### Treatment (or lack of) phase and living with illness

#### The impact of living with a life-shortening condition

Some patients described a shift in identity as they came to terms with their diagnosis, and also as they adjusted to life with a life-shortening condition. Particularly when it impacted their physical appearance:

*I mean, you wonder who you are, don’t you? When you’re given a diagnosis and it’s terminal, you wonder who this person is and how you’re going to be because it’s a new experience; you have no idea how you’re going to face it or how you’re going to explain yourself and the story that you tell to people.* (Patient, Focus Group 6)

As well as the shift in identity, patients expressed a wide range of emotions following their diagnosis. One of the key findings identified was this notion of living with the unknown. Patients reported that they did not know how their illness would evolve, nor how much time they had left:

*Once I’ve had cancer, I wouldn’t like celebrate five years cancer free because you just don’t know. You just don’t know when it might come back. So, I just used to think, “Well, I’ve had it and keep up the appointments …” you just carry on.* (Patient, Focus Group 5)

Patients went on to describe how living with their illness affected their everyday life and how they had to adapt as they were unable to do the things they had done previously:

*Obviously a lot of things I struggle with. And the most annoying thing is … it’s the frustration.* (Patient, Focus Group 1)

#### Challenges to patient and family carer relationships

In most cases, family members became the main carers for patients with a life-shortening condition. Several patients described how they kept many of their feelings and emotions from their loved ones in an attempt to protect them from knowing the true extent of the impact of their illness:

*When I was really down and really very unwell, I just withdrew from almost everybody really because I don’t want to be this moaning patient who’s obsessed with all the bits of everything falling off and going wrong. But … the community nurse was a support and I think I’ve been referred to the bereavement team here for counselling. And I would feel happier talking to them almost in a way because they’re being paid to listen rather than burdening friends and family with, with what happens to me.* (Patient, Focus Group 5)

As the quotation highlights, some patients were grateful when they were offered counseling to offload to a professional rather than a family member. This was, however, not always offered, or easy to access:

*I hardly tell my wife anything ‘cause I’m frightened – I’m frightened to tell her ‘cause if she knew how bad I feel, she would feel really awful. She would feel that she had done something wrong. And it’s just like the burden of, of what you carry as a patient, really. But there are people there that can help. But they’re [healthcare providers] not very good at sending the messages out really, to say what there is available.* (Patient, Focus Group 5)

Taking on the role of carer impacted individuals in several ways: individuals expressed worry for the patient, but they also worried in case they were no longer able to look after them. Some family members expressed denial, or frustration, while others focused on caring for patients in the best way they could, whatever the circumstances:

*It’s really worrying as a carer though, isn’t it, watching somebody not eat. And as a carer you want to feed, you want to nourish, you want to make somebody feel better and food is often part of that. So, that’s really difficult as a carer.* (Wife of Patient, Focus Group 6)

Some family members felt well supported by healthcare services and this helped them to cope during difficult and challenging times. For example, some individuals spoke about how visits from community nurses allowed them some respite. Nevertheless, others were unable to access this support or did not know it was available. The following section examines this in more detail.

#### A sense of abandonment when care is not provided/cannot be accessed

There were times when bereaved family members reflected on their experiences of caring for their loved ones at home and described times when they felt very alone. This was exacerbated when access to out-of-hours and community care was challenging, or if they did not know it was available. There were accounts of families not knowing who to call for general support when distressed and either not being able to get through to service providers, or not hearing back from them. There were also instances where patients were sent home from hospital without any support in place or medication:

*She [doctor] didn’t tell us we were supposed to have an end-of-life team come to visit us and he [husband] was discharged home, no medication for his pain. He was told that the oncology department would ring us the day he came home to fix up an appointment … So, it was Friday. I couldn’t get hold of the oncology team. I couldn’t get hold of the urology team. The hospice team never contacted us.* (Bereaved Wife, Focus Group 2)

Some of the participants used the word “abandonment” to describe their experiences:

*And I thought … “God. You know. This is healthcare. This is health sort of abandonment.”* (Bereaved Wife, Focus Group 4)

Some patients reported being left in a state of limbo when there were no treatments available for their illness, or if they declined treatment. This left some patients and their families feeling discarded and/or isolated as they had little or no contact with healthcare providers/professionals. One participant said:

*I’m in my fifth year of having cancer. Tried various treatments and then was passed from the hospital because there was no treatment available, and I only had a few weeks to live last July. So, I’ve not had any real medical attention since then because I’m too well for the… hospice really and I’m too ill for the hospital apparently. So, I’m in – been in limbo for nearly a year now.* (Patient, Focus Group 6)

#### A lack of personalized care

Both patients and families described a lack of person-centered care, particularly in hospital. There were accounts where healthcare professionals showed a lack of empathy and compassion or seemed unable to go “above and beyond” the provision of essential medical care. The lack of face-to-face appointments in some areas also contributed to this, as well as a lack of continuity of staff. Therefore, patients did not feel like professionals got to know them and/or their preferences:

*When I was a patient in the day hospice I felt listened to. When you go to the hospital, the part of the hospital where the cancer patients go is packed full of people and it’s just like a conveyor belt really. You know, you have your forms, you go for your blood test. They ring you to say if your treatment’s going ahead or not or … And, and you don’t really have any control. Everything is led by – obviously by the consultant but then it filters out and people just see it as a job.* (Patient, Focus Group 5)

### The very end-of-life

#### Feeling prepared for death

There was some discussion around patients’ personal feelings toward death and dying. Some patients and family members had begun preparing, while others had not. Some individuals felt that more conversations with healthcare professionals around preparing for death were needed. They felt this would have been helpful and could have potentially eased some of their fears by helping them to understand what to expect:

*There was a young girl at the day hospice in her early 40s and she was terrified of dying … A patient came round to join us and we all got talking and this, this young girl was listening to what the patient was saying and her experience and the next week, the patient didn’t come, she’d died. But the young girl said, “… She really helped me ‘cause she talked me through things and explained what the hospice was like and explained about pain control and you know, what went on.” And I thought, “That shouldn’t be left to chance really.” … That young girl should somehow be able to access information that, that she needs.* (Patient, Focus Group 5)

#### Traumatic experiences of death

Some bereaved family members recounted traumatic experiences as their loved ones passed away, particularly if they were in pain at the end of their life. This trauma was heightened when they felt abandoned by healthcare providers and when the support they needed was not in place:

*The emotional trauma. It was those last few hours. You tear yourself apart. For the whole year, I kept thinking, “Perhaps if I’d done something a bit different. Perhaps if I had done this. Perhaps if I had done that. I could have made him [husband] more comfortable and could have …” you know. “Perhaps if I hadn’t called the doctor that afternoon and…” And I just couldn’t get through it. Which is why it’s taken me so long to come through to the hospice, you know, ‘cause I just couldn’t get through it.* (Bereaved Wife, Focus Group 2)

### Post-death of loved one

#### Adjusting to the loss of a loved one

As well as the trauma caused by the negative experiences of the death of a loved one, several bereaved family members described how they had struggled in the period immediately after death, and over the longer-term. Some experienced an adjustment in identity as they were no longer a “carer,” while others missed the companionship provided by their partners:

*But also I think perhaps more so when you’re older maybe – you’ve lost your life; you’ve lost your identity … My husband and I, we worked together; we did everything together; we have no children. We worked long, long hours, so when he went I had no social structure. I had no interests really.* (Bereaved Wife, Focus Group 2)

Some felt there was a need for more bereavement support. In some cases, individuals had been offered this soon after the death, which they felt was too soon. Others had not been offered any support and/or did not know how to access it:

*There isn’t any support out there and … although I had the sort of benefit of being a retired professional health person, when – it’s when it’s your, your absolute reason for living – you lose all that professional veneer and you lose that kind of, you know, “I can do this.” … And I became, you know, the gibbering wreck. And I lost my mind. I can tell you now, I did. I was mentally very unwell. But there was nobody.* (Bereaved Wife, Focus Group 4)

## Discussion

This study provides insights into patient, family carer, and bereaved family member experiences of palliative and EoL care in South-West England. It has highlighted the complex and multifaceted needs of patients and families at different points in their journey from diagnosis to the time after death. It has shown things that families found helpful and has drawn attention to areas which could be improved.

Communication with healthcare professionals emerged as an important topic. As previous research has shown, patients and families often report poor experiences of communication from healthcare professionals (Anderson et al. 2019) and there was a clear need for improvement in this area. Some participants felt that more openness and honesty about their situation would have been beneficial, particularly around how an illness may potentially evolve and also around death and dying. This would enable them to know what to expect and to feel more prepared for the future (Herbert et al. 2009; Steinhauser et al. 2015). Other studies have shown that healthcare professionals often find EoL communication challenging and sometimes wait for patients or families to initiate such conversations (Anderson et al. 2019; Nedjat-Haiem et al. 2017). Some families felt that more education and training for healthcare professionals was required to help them improve in this area. Participants felt improvement was needed in the way life-shortening diagnoses were delivered and also felt that the delivery of information around prognosis and care should be tailored to each individual family to allow them time to process and cope with information linking to the need for personalized care (see also Engel et al. 2023). As Wenrich et al. (2003) observe, although previous research has illustrated that healthcare needs to attend to the “whole person,” few studies have addressed exactly how this can be achieved and what the emotional needs of patients are. As well as tailoring the delivery of information to each patient and their family, participants felt that they should have a choice about what information they receive and when. This could empower individuals and give them more of a sense of feeling in control (Reeve et al. 2012).

Participants also emphasized the importance of emotional support and how this would be valued by some – again, preference was dependent on the individual – both pre- and post-death (Milberg et al. 2008; Wenrich et al. 2003). Some patients who had accessed forms of emotional support felt they had benefited from it, especially those who had not shared their true feelings with family members in an attempt not to burden them. Some bereaved family members also shared how bereavement support had helped them to cope after a loved one’s death. As previous research has shown, bereavement support is effective in reducing grief, depression, and anxiety (Kustanti et al. 2021). However, the findings have illustrated that bereavement support needs to be made available to people when they feel they need it, and not just in the immediate period after death. Clearer signposting would also be beneficial so that individuals know where and how they can access it. Continuity of staff providing bereavement support in some hospices was found to be helpful because staff understood the circumstances leading to bereavement and had built rapport and trust with patients and families (Harrop et al. 2020).

Findings also demonstrate the need for adequate provision of out-of-hours and community care. In particular, there was a need for patients and their families to be told what care is available, how it can be accessed, by whom, and in which scenario. Wider research has illustrated the barriers experienced by palliative care patients and their carers when accessing out-of-hours care and that such services need to be appropriately resourced and made more accessible (Worth et al. 2006). This was particularly important for our participants as they recounted the emotional distress and sense of abandonment they felt when they were unable to access the support they needed (see also Mason et al. 2022).

## Study limitations

There were several strengths to this study, particularly in that it offered individuals with a life-shortening condition the opportunity to participate in research – something which is often seen as challenging and faces many barriers (Kendall et al. 2007). Bringing participants together to talk in groups also appeared to be beneficial and some individuals expressed that they had got a lot out of the experience of talking to others in a similar situation (see also Jones and Neil-Urban 2003). There are also some limitations that should be considered. First, the sample was quite small (*n* = 18) and therefore would not be fully representative of the wider population. Second, the sample was not diverse in terms of some demographics, particularly ethnicity – although the South-West generally is not an ethnically diverse area (Gov.uk 2022). Future research should consider the experiences of those from different ethnic backgrounds and those experiencing inequalities. As participants were recruited by hospices, they had received some specialist palliative care/contact. Future work should also consider the experiences of those who have not received this. Finally, not all patients and family members shared details of the patients’ diagnosis, so it was not always clear how this affected the care they received. There are many types of conditions that were not included in the study that may have additional unmet needs. The majority of patients who participated in the study and shared their diagnosis had a cancer diagnosis; however, it is important to investigate the experiences of those with other life-shortening conditions.

## Implications for future practice

The findings of this study identify the following areas for professionals and policy makers to consider: (i) there is a need for more education and training to improve the way healthcare professionals communicate with patients and families, particularly when sharing the diagnosis of a life-shortening condition; (ii) care and support must be personalized so that patients feel empowered and have a say in their treatment/care; (iii) emotional support should be made available and clearly signposted throughout the patient and family journey; (iv) out-of-hours and community care should be in place so that patients and families do not feel a sense of abandonment.

Hospices, including those involved in this study, are in a strong position to support these areas of practice development. However, to do this there would need to be further consideration given to how this future support is commissioned and funded (All Party Parliamentary Group 2024).

## Conclusion

This study has described how palliative and EoL care in South-West England are perceived by patients and their families. It has highlighted some of the challenges that patients with a life-shortening condition and their families experience and the subsequent emotions. It has made recommendations to help improve these experiences. It has also shown the benefits of participating in focus groups for individuals. Future research is needed, however, to explore the experiences of more diverse groups and those with other conditions beyond cancer.

## Supporting information

Kallis et al. supplementary material Kallis et al. supplementary material

## Supplementary material

The supplementary material for this article can be found at https://doi.org/10.1017/S1478951525000057.
