# Supplementary material for: Exploring the challenges experienced by patients and families using palliative and end-of-life care services: A qualitative focus group study
Source: Palliat Support Care. 2025 Mar 3;23:e70. doi: 10.1017/S1478951525000057 (PMC13166391; doi:10.1017/S1478951525000057)
Supplement: Kallis et al. supplementary material [file S1478951525000057sup001.docx]

## Appendix 1: Focus Group Guide

- Welcome/introductions
- Discuss aim of focus group
- Discuss ground rules for the session (e.g. focus is on hearing their thoughts/experiences, everyone treated with respect etc./confidentiality/ability to leave at any time).

Everyone introduce themselves and share why they have decided to come to the focus group.

**Questions**

1. What are your particular experiences of being near the end of life/ palliative/end of life care?
2. What has been most important to you? The most helpful?
3. What have been the biggest challenges?
4. Have you had any experiences of research?
5. Having a few moments to think - about your experiences and more generally - are there key areas we need to know more about?
6. What do you think your priority for research would be? Would you be able to share it with the group?
7. What are your thoughts about the other priorities discussed here?
8. (Priorities listed on the board) Can you list the priorities from most to least important?
9. Is there anything else anyone would like to add about the research priorities or anything else which is important?
